# Changes in Antimicrobial Resistance in Bloodstream Infections During the COVID-19 Pandemic—A Retrospective Study from a Tertiary Care Hospital

**DOI:** 10.3390/microorganisms14102269

**Published:** 2026-10-06

**Authors:** Rodica Stela Lumpan, Daniela Tălăpan, Alina Maria Borcan, Mihai Stoica, Mihai Octavian Dan, Alexandru Rafila, Victoria Aramă

**Affiliations:** 1 Faculty of Medicine, ‘Carol Davila’ University of Medicine and Pharmacy, 021021 Bucharest, Romania; rodica-stela.lumpan@drd.umfcd.ro (R.S.L.);; 2 Ploiesti Pediatric Hospital, 100336 Ploiesti, Romania; 3 ‘Prof. Dr. Matei Bals’ National Institute of Infectious Diseases, 021105 Bucharest, Romania; 4 Faculty of Marketing, Bucharest University of Economic Studies, 010374 Bucharest, Romania

**Keywords:** antimicrobial resistance, invasive infections, blood cultures, COVID-19, carbapenem resistance

## Abstract

The COVID-19 pandemic led to major repurposing of healthcare settings, especially Infectious Diseases hospitals, by redirecting care towards SARS-CoV-2-infected patients. However, frequent bacterial infections overlapping the viral background led to changes in antimicrobial resistance profiling among patients. This study aims to assess isolates from invasive infections and their antimicrobial susceptibility patterns in pre-pandemic and pandemic periods. Positive blood cultures from patients hospitalized in our Institute between January 2019 and September 2022 were assessed in terms of antimicrobial susceptibility, following sample processing as per laboratory protocol, with the use of the automated Bactalert^®^3D system, MALDI-TOF^®^ MS and microscopy for bacterial identification. Antimicrobial susceptibility testing was performed using the Vitek2Compact^®^ and Micronaut-S^®^ systems. Phenotypic enzyme production testing was assessed using the Resist-5 OKNVI kits. All data were subsequently digitalized, and statistical analysis was performed. A total of 932 non-duplicated bacterial and fungal strains were analyzed, with the most commonly isolated organisms being *Staphylococcus aureus* (*n* = 173), followed by *Escherichia coli* *(n* = 156), *Acinetobacter baumannii* (*n* = 95), and *Klebsiella pneumoniae* (*n* = 89). Resistance rates to multiple antimicrobials increased substantially amongst *Klebsiella pneumoniae* and *Acinetobacter baumannii*. Methicillin-resistant *Staphylococcus aureus* isolates followed a national decrease trend during the pandemic period; however, an increasingly resistant profile to macrolides may be attributable to the excessive and often unjustified use of Azithromycin during the pandemic period. The COVID-19 pandemic was accompanied by a shift in bacterial profile of invasive infections amongst our cohort, illustrating the dynamic nature of antimicrobial resistance in an Infectious Diseases hospital during those times, underscoring the complex interplay between the COVID-19 pandemic and antibiotic resistance patterns, and highlighting the need for ongoing surveillance and tailored antimicrobial stewardship efforts in clinical practice.

## 1. Introduction

The acute respiratory syndrome caused by the SARS-CoV-2 virus has been one of the major public health challenges globally since the onset of the pandemic in late 2019, burdened with high morbidity and mortality. Although various clinical presentations have been described, lung injuries have remained a common manifestation, represented by diffuse alveolar destruction that overlaps with the definition of acute respiratory distress syndrome, characterized by epithelial and endothelial injury, with alveolar–capillary barrier impairment and secondary edema. Additionally, a characteristic feature of COVID-19 is the presence of coagulation abnormalities with the secondary appearance of microthrombi in pulmonary capillaries, with pulmonary embolism contributing significantly to mortality [1,2].

In clinical practice, patients with severe forms of the disease undergo invasive procedures such as mechanical ventilation and are treated with corticosteroids and antibiotics to prevent and treat bacterial superinfections [2,3]. These patients, often presenting with comorbidities and advanced age, become vulnerable to superinfections with multidrug-resistant bacteria [3]. The overconsumption of antibiotics and disinfectants is added to these factors [4].

Regarding the proportion of patients with bacterial superinfections associated with COVID-19, it is estimated that between 1 and 10% of patients have presented bacterial superinfections, and at least 45% of the total number of patients have received antibiotic therapy [5]. Although the World Health Organization contraindicates empirical antibiotic therapy in its guidelines, antibiotics have been used to varying extents in different locations globally since the beginning of the pandemic [6]. This phenomenon may have accentuated the selection pressure on bacteria, leading to an increase in global antibiotic resistance levels.

In the case of Romania, official reports yield high rates of both consumption and resistance of antimicrobials, with the World Health Organization (WHO) and European Center for Disease Prevention and Control (ECDC) reporting some of the highest antimicrobial resistance rates in Europe, mostly in the cases of pathogens isolated from invasive infections, such as *Klebsiella pneumoniae*, *Pseudomonas aeruginosa* or *Acinetobacter baumannii* [7,8,9,10,11].

This study aims to characterize antimicrobial resistance patterns in pathogens isolated from positive blood cultures of patients admitted to a tertiary care, Infectious Diseases hospital in Bucharest, Romania, aiming to unveil the dynamics of infections before and during the COVID-19 pandemic, illustrating the role this public health crisis played in modifying resistance rates and pathogen isolation patterns.

## 2. Materials and Methods

### 2.1. Study Design

This retrospective study was conducted in the Clinical Microbiology Laboratory of the ‘Prof. Dr. Matei Balș’ National Institute of Infectious Diseases (NIID) in Bucharest, Romania. Non-duplicate pathogenic strains isolated from positive blood cultures from patients hospitalized in the NIID between 1 January 2019 and 30 September 2022 were included for analysis. Also, the coagulase-negative staphylococci (one per patient), if the same species and the same antibiotype were isolated from at least 2 bottles originating from 2 different sets of blood cultures, following consultation with the clinician, were included in the study. Starting 1 March 2020, the hospital functioned exclusively for SARS-CoV-2-infected patients; thus, the cohort has been divided into two study lots—pre-pandemic (1 January 2019–29 February 2020) and pandemic (1 March 2020–30 September 2022).

Exclusion Criteria: Positive blood cultures from patients whose clinical and biological status does not indicate possible sepsis, and from whom the isolated bacteria are frequently contaminants (e.g., *Bacillus* spp.), were excluded. Positive blood cultures with commensal bacteria from the normal skin flora that have entered the blood culture bottle due to inadequate skin disinfection were also excluded.

### 2.2. Bacterial Culture

The investigation of bacterial pathogens in clinical specimens was carried out at the request of the Infectious Disease physician, and the analysis of specimens followed standard methods of isolation, identification, and antibiotic sensitivity testing, according to working procedures, in accordance with the EUCAST standard. Samples were processed and analyzed in the Clinical Microbiology Laboratory according to specific procedures for sample collection, transport, and reception. Primary processing of blood cultures was performed using the automated Bactalert^®^3D system (bioMérieux S.A., Marcy-l’Etoile, France). Positive blood cultures were inoculated afterwards on Columbia sheep blood agar (COS, ThermoScientific™—Oxoid, Wesel, Germany).

### 2.3. Strain Identification

All bacterial strains were identified using a Matrix-Assisted Laser Desorption Ionisation Time-of-Flight Mass Spectrometry (MALDI-TOF MS) Biotyper (Bruker Daltonik GmbH, Bremen, Germany), which characterizes bacterial proteins in whole-cell extracts, corroborated by microscopic analysis. Bacterial spectra were analyzed using the Biotyper software version 4.1.

### 2.4. Antimicrobial Susceptibility Testing and Quality Control

Antimicrobial susceptibility testing (AST) was conducted using automated systems for measuring minimum inhibitory concentrations (MICs)—VITEK^®^ 2 Compact System (bioMérieux S.A., Marcy-l’Etoile, France) and MICRONAUT-S (Bruker Daltonik GmbH, Bremen, Germany)—followed by interpretation according to the EUCAST (European Committee on Antimicrobial Susceptibility Testing) guideline versions 9.0, 10.0, 11.0 and 12.0, respectively. All Gram-negative strains tested on VITEK were verified for susceptibility to Colistin using UMIC Colistin (Bruker Daltonik GmbH, Bremen, Germany). Carbapenemase-positive strains were tested for susceptibility to Ceftazidime/Avibactam using *E*test strips (Marcy l’Etoile, France, bioMérieux), according to the manufacturer’s procedure. The ATCC strains *E. coli* 25922, *P. aeruginosa* 27853, *S. aureus* 29213, *E. faecalis* 29212 and *S. pneumoniae* 49619 were used for AST quality control according to the EUCAST guideline.

### 2.5. Enzyme Production Testing

Phenotypic testing for enzyme production (extended-spectrum beta-lactamase and carbapenemases) was performed following the EUCAST guideline on detection of resistance mechanisms version 2.0, using lateral flow assays. NG Test/CTX-M Multi (NG Biotech, Guipry, France) was performed to identify CTX-M production, and Resist-5 O.K.N.V.I. (Coris BioConcept, Belgium, Gembloux, Belgium) to detect OXA-48-type, KPC, NDM, VIM and IMP carbapenemases. Enzyme production testing was performed selectively, on isolates showing reduced susceptibility or resistance to at least one carbapenem on phenotypic testing, according to the EUCAST guidelines for detection of resistance mechanisms and specific resistances of clinical and/or epidemiological importance version 2.0 [12].

### 2.6. Data Storage and Statistical Analysis

All data were subsequently digitalized into a project-assigned Microsoft Excel database, with base statistical analysis performed using Microsoft Excel version 16.66.1 2022 (Microsoft Corp., Redmond, WA, USA) and IBM SPSS Statistics 26.0 software package (IBM Corp., Armonk, NY, USA). A two-tailed Fisher’s exact test was performed to determine the difference in proportions between the two sublots studied and results were considered statistically significant at *p* value < 0.05, after the Benjamini–Hochberg false discovery rate (FDR) correction [13] was applied across all organism–antimicrobial comparisons, and only those corresponding to an FDR-adjusted *q*-value < 0.05 were considered statistically significant. Given the large number of organism–antimicrobial comparisons performed, for every organism–antimicrobial combination, the exact number of isolates tested, the number of resistant isolates, the 95% confidence interval (Wilson score method), and the Benjamini–Hochberg FDR *q*-value are reported in Supplementary Table S1.

## 3. Results

### 3.1. Bacterial Strains

**Figure 1:**
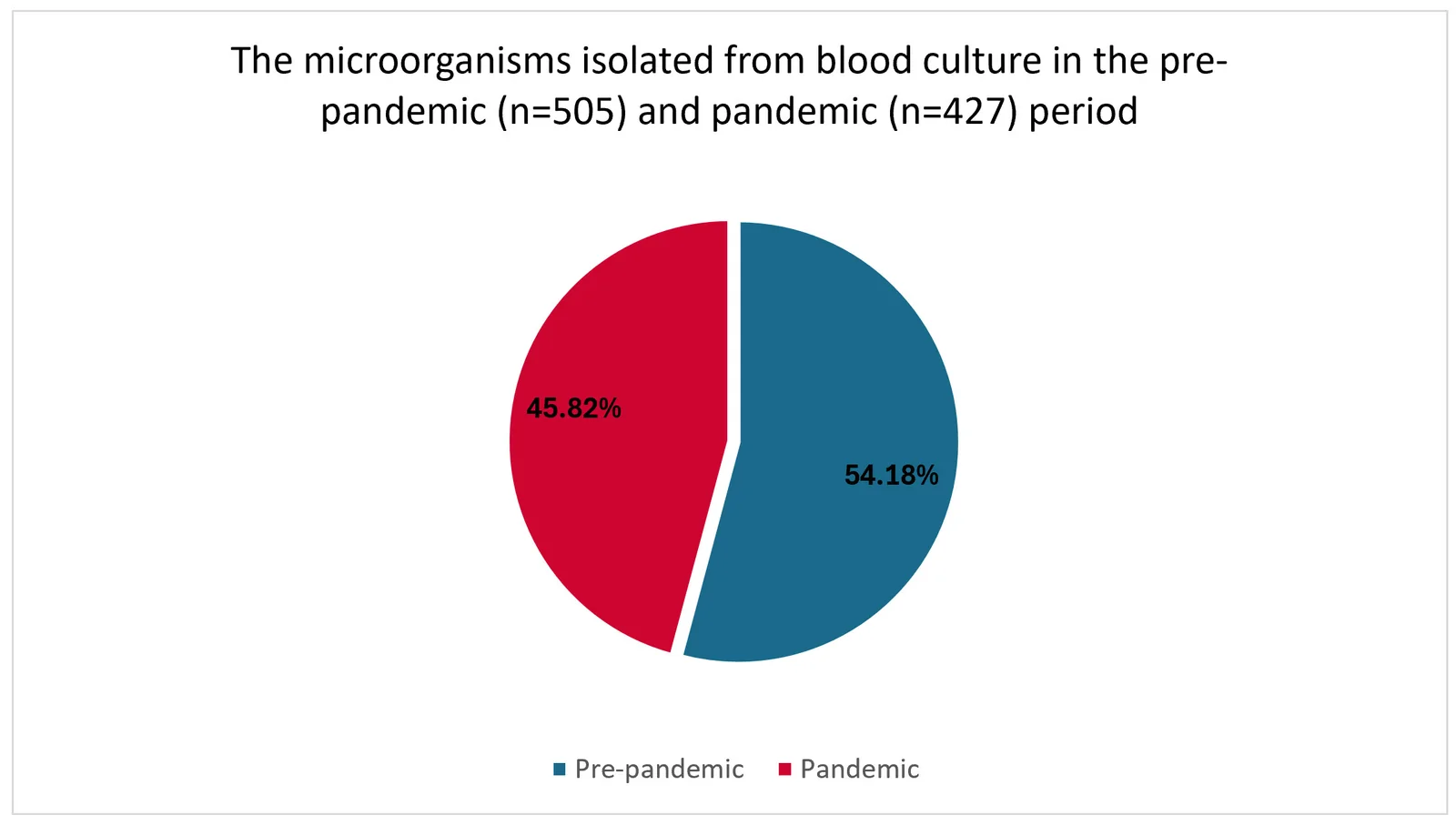
The bacteria isolated from blood culture during the study period.

There were 932 isolated non-duplicate bacterial strains from patients admitted to NIID during 1 January 2019–30 September 2022. The proportion of microorganisms isolated from patients with COVID-19 infection was non-significantly smaller than the ones isolated from patients with sepsis admitted in the NIID in the pre-pandemic period (Figure 1).

The details regarding bacteria isolated from blood culture during the study period are found in Table 1. The microorganisms isolated from patients admitted into the Infectious Disease wards significantly decreased during the pandemic period (Table 2), but at the same time there was an increase in the microorganisms isolated from patients admitted into Intensive Care Unit (ICU) and from the ones arriving at the Emergency Room (ER).

Looking closely at the type of microorganisms isolated in the pandemic period in comparison with the ones isolated before the pandemic period, there is evidence that the number of glucose fermenters (bacteria from Enterobacterales order) significantly decreased (and also the streptococci), while the number of glucose non-fermenters (especially *Acinetobacter baumannii*) increased (Table 3). Also, there were no yeasts isolated from blood cultures from patients admitted in the NIID in the pre-pandemic study period, while these were isolated during the pandemic period (including the first strain of *Candidozyma auris*).

### 3.2. Antimicrobial Susceptibility Testing Results

The exact number of isolates tested against each antimicrobial agent, by organism and period, is provided in Supplementary Table S1.

#### 3.2.1. Gram-Negative Rods

**Figure 2:**
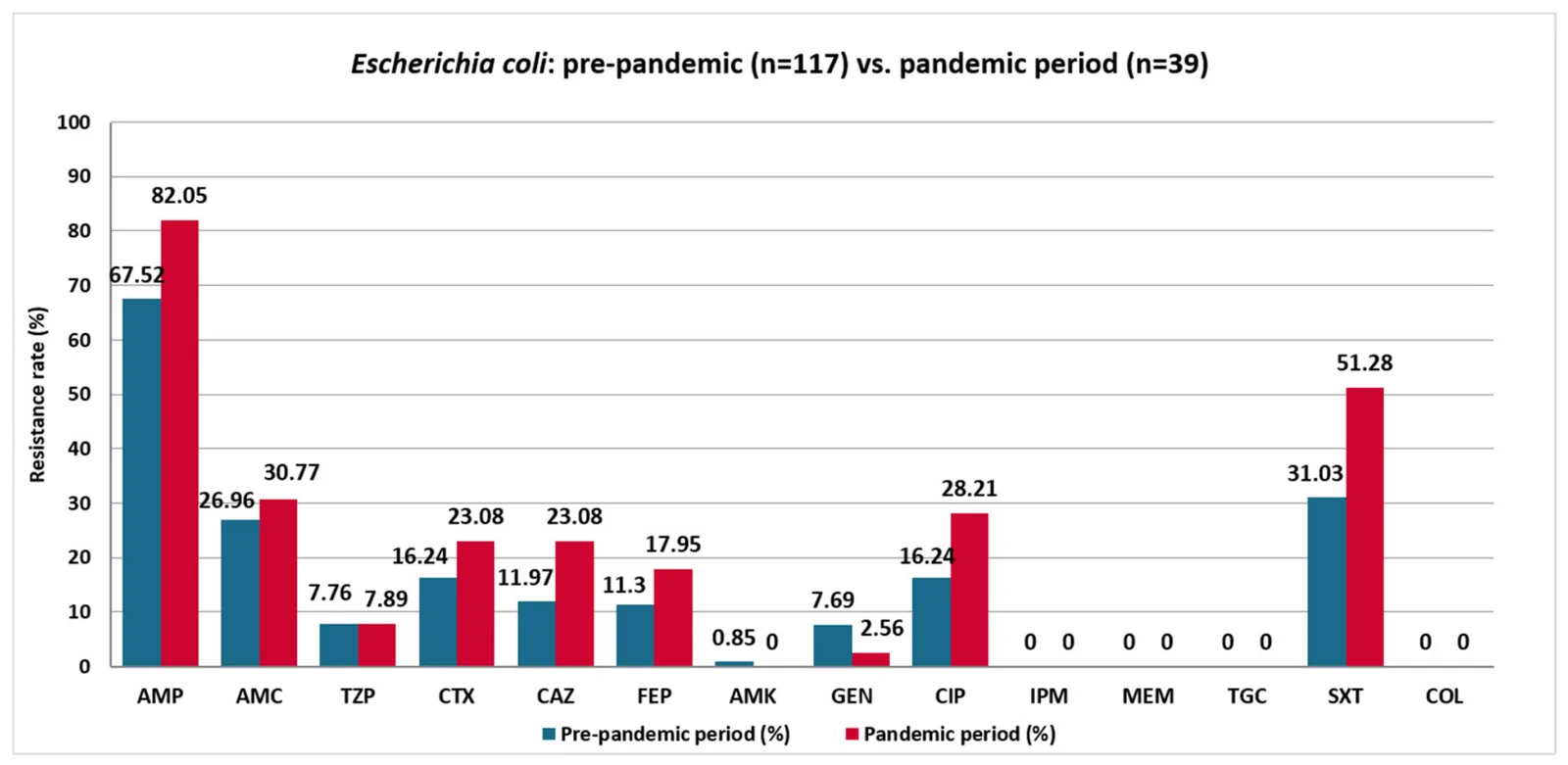
Antimicrobial resistance in *E. coli*. Note: Values shown are exact R%. AMP—Ampicillin, AMC—Amoxicillin/Clavulanate, TZP—Piperacillin/Tazobactam, CTX—Cefotaxime, FEP—Cefepime, CAZ—Ceftazidime, AMK—Amikacin, GEN—Gentamicin, CIP—Ciprofloxacin, IPM—Imipenem, MEM—Meropenem, TGC—Tigecycline, SXT—Trimethoprim/Sulfamethoxazole, COL—Colistin.

*Escherichia coli* was tested against 14 antimicrobials, and the comparison between pre-pandemic and pandemic periods revealed a predominantly non-significant pattern, with resistance rates increasing numerically for most agents but without reaching statistical significance after correction for multiple comparisons. Resistance to Imipenem, Meropenem, Tigecycline, and Colistin remained at 0% across both periods, indicating sustained susceptibility and confirming the continued utility of carbapenems and last-line antimicrobials (Figure 2).

**Figure 3:**
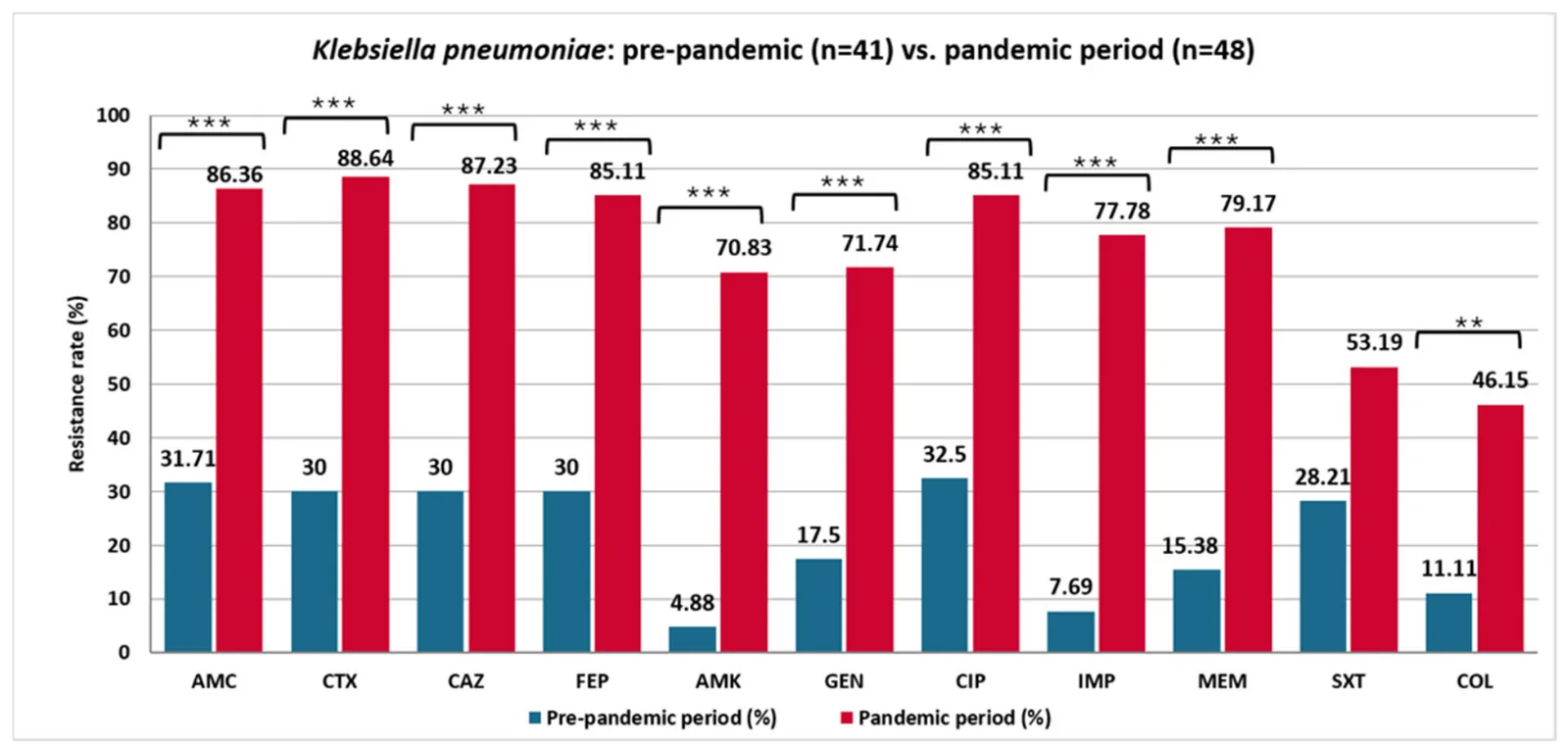
Antimicrobial resistance in *Klebsiella pneumoniae*. Note: Only comparisons remaining statistically significant after Benjamini–Hochberg correction for multiple comparisons (FDR-adjusted *q* < 0.05; see Supplementary Table S1) are marked with brackets. Asterisks indicate the magnitude of the unadjusted Fisher’s exact test *p*-value (** *p* < 0.01, *** *p* < 0.001), shown for descriptive purposes. Values shown are exact R%. Antibiotic abbreviations: AMC—Amoxicillin/Clavulanate, CTX—Cefotaxime, FEP—Cefepime, CAZ—Ceftazidime, AMK—Amikacin, GEN—Gentamicin, CIP—Ciprofloxacin, IPM—Imipenem, MEM—Meropenem, SXT—Trimethoprim/Sulfamethoxazole, COL—Colistin.

For *Klebsiella pneumoniae*, the comparison between pre-pandemic and pandemic periods revealed a predominantly unidirectional shift toward increased resistance during the pandemic period, statistically significant after FDR correction for 10 of the 11 antibiotics evaluated. Statistically significant increases were observed for: Amoxicillin/clavulanate 31.71% → 86.36%, *p* < 0.001; Cefotaxime 30.00% → 88.64%, *p* < 0.001; Ceftazidime 30.00% → 87.23%, *p* < 0.001; Cefepime 30.00% → 85.11%, *p* < 0.001; Amikacin 4.88% → 70.83%, *p* < 0.001; Gentamicin 17.50% → 71.74%, *p* < 0.001; Ciprofloxacin 32.50% → 85.11%, *p* < 0.001; Imipenem 7.69% → 77.78%, *p* < 0.001; Meropenem 15.38% → 79.17%, *p* < 0.001; and Colistin 11.11% → 46.15%, *p* < 0.01 (Figure 3).

For other Enterobacterales (*Enterobacter* spp. and other miscellaneous Enterobacterales, combined), no statistically significant differences in resistance rates were observed for any of the nine antibiotics tested between the pre-pandemic and pandemic periods (Supplementary Table S1).

**Figure 4:**
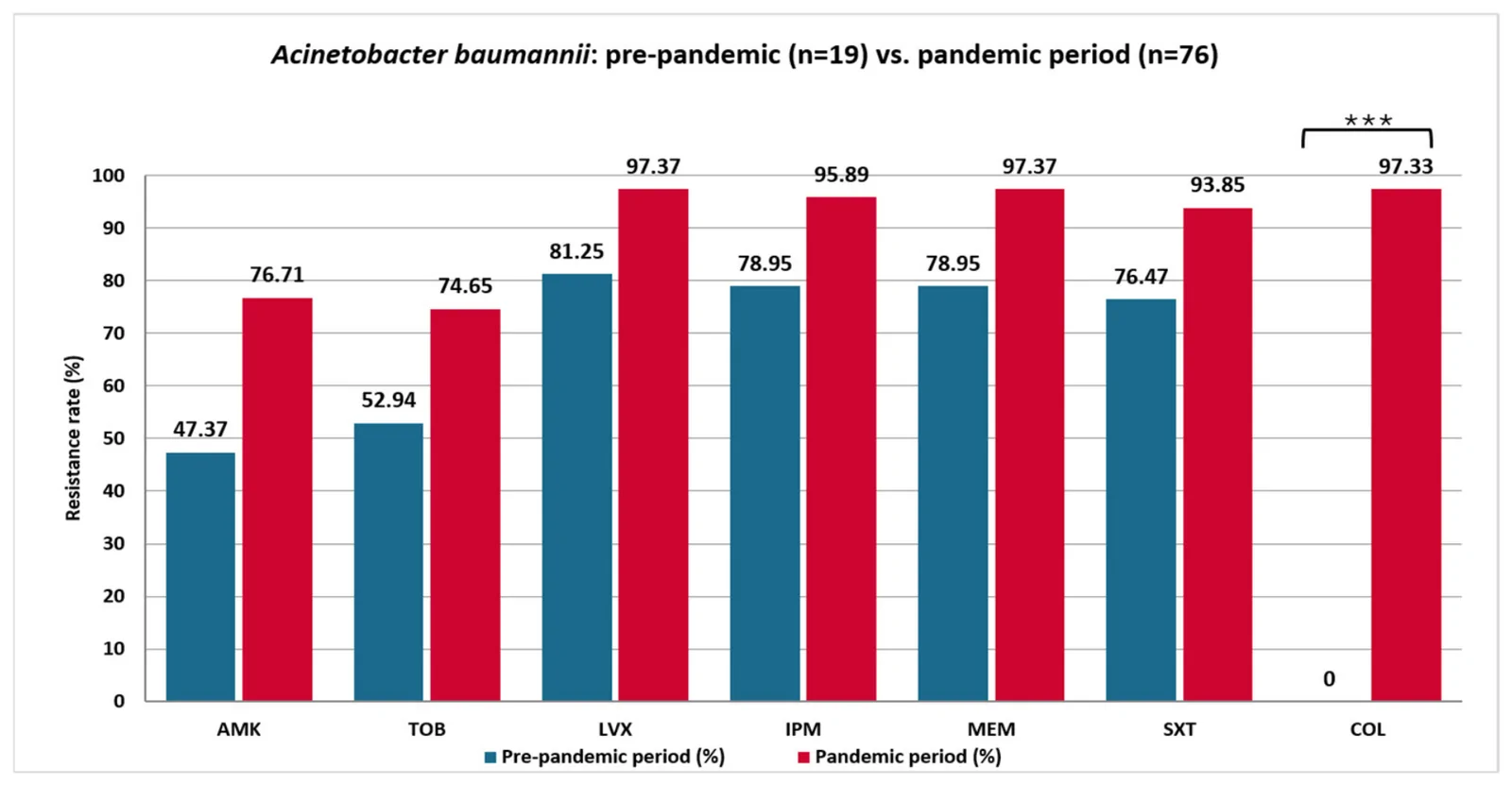
Antimicrobial resistance in *Acinetobacter baumannii*. Note: Only comparisons remaining statistically significant after Benjamini–Hochberg correction for multiple comparisons (FDR-adjusted *q* < 0.05; see Supplementary Table S1) are marked with brackets. Asterisks indicate the magnitude of the unadjusted Fisher’s exact test *p*-value (*** *p* < 0.001), shown for descriptive purposes. Values shown are exact R%. Antibiotic abbreviations: AMK—Amikacin, TOB—Tobramycin, LVX—Levofloxacin, IPM—Imipenem, MEM—Meropenem, SXT—Trimethoprim/Sulfamethoxazole, COL—Colistin.

Across the seven antibiotics evaluated in *Acinetobacter baumannii* blood cultures, the comparison between pre-pandemic and pandemic periods revealed a unidirectional shift toward increased resistance during the pandemic period (Figure 4), but only the increase in Colistin resistance remained significant after Benjamini–Hochberg correction for multiple comparisons (*p* < 0.001).

For *Pseudomonas aeruginosa*, no statistically significant differences in resistance rates were observed for any of the nine antibiotics tested between the pre-pandemic and pandemic periods (Supplementary Table S1).

#### 3.2.2. Gram-Positive Cocci

**Figure 5:**
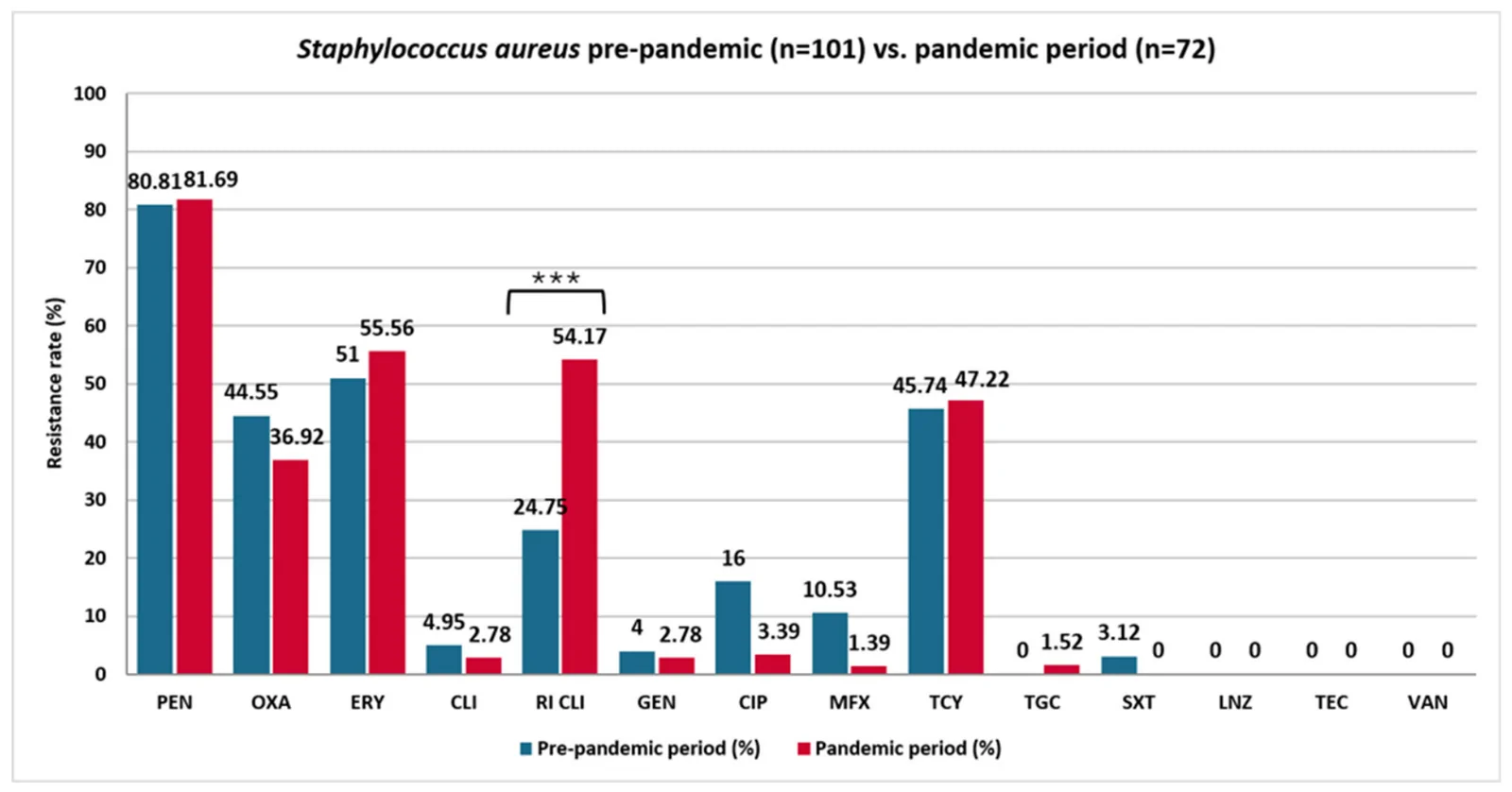
Antimicrobial resistance in *Staphylococcus aureus*. Note: Only comparisons remaining statistically significant after Benjamini–Hochberg correction for multiple comparisons (FDR-adjusted *q* < 0.05; see Supplementary Table S1) are marked with brackets. Asterisks indicate the magnitude of the unadjusted Fisher’s exact test *p*-value (*** *p* < 0.001), shown for descriptive purposes. Values shown are exact R%. Antibiotic abbreviations: PEN—Penicillin, OXA—Oxacillin, ERY—Erythromycin, CLI—Clindamycin, RI CLI—Clindamycin Inducible Resistance, GEN—Gentamicin, CIP—Ciprofloxacin, MFX—Moxifloxacin, TCY—Tetracycline, TGC—Tigecycline, SXT—Trimethoprim/Sulfamethoxazole, LNZ—Linezolid, TEC—Teicoplanin, VAN—Vancomycin.

Across the 14 antibiotics evaluated in *Staphylococcus aureus* blood cultures (pre-pandemic *n* = 101; pandemic *n* = 72), comparison between periods revealed a mixed pattern of resistance changes, with statistically significant increases for Clindamycin inducible resistance—24.75% → 54.17%, *p* < 0.001 (Figure 5).

Across the antibiotics evaluated in coagulase-negative staphylococci blood isolates (pre-pandemic *n* = 36; pandemic *n* = 42), the comparison between periods revealed a predominantly non-significant pattern, with resistance decreasing numerically for most agents but without reaching statistical significance.

For *Enterococcus faecalis* and *Enterococcus faecium*, analyzed separately by species, no statistically significant differences in resistance rates were observed for any of the five antibiotics tested between the pre-pandemic and pandemic periods.

For *Streptococcus pneumoniae*, no statistically significant differences in resistance rates were observed for any of the 12 antibiotics tested between the pre-pandemic and pandemic periods (full denominators, percentages, and FDR-corrected *q*-values for all three organism groups above are reported in Supplementary Table S1).

### 3.3. Enzyme Production Testing Results

Among *E. coli* isolates from blood cultures, the ESBL-positive strains decreased from 12.82% (pre-pandemic *n* = 15/117) to 10.26% (pandemic, *n* = 4/39). There was no carbapenemase-positive *E. coli* strain. For *K. pneumoniae* strains, both the ESBL (*n* = 11/41 pre-pandemic, *n* = 35/48 pandemic)- and carbapenemase-producing strains (*n* = 7/41 pre-pandemic, *n* = 39/48 pandemic period) increased in the pandemic period compared with the pre-pandemic one, the most frequently detected carbapenemase in the pandemic period being NDM (53.85%) (Table 4). *K. pneumoniae* carbapenemase-positive strains were tested for Ceftazidime/Avibactam susceptibility throughout the study period, with the following results: KPC-positive strains were all susceptible and NDM (alone or in combination)-positive strains were all resistant.

## 4. Discussion

The present retrospective study assessed the etiology and antimicrobial resistance (AMR) profiles of bacteria isolated from the bloodstream at the ‘Prof. Dr. Matei Balș’ National Institute of Infectious Diseases, Bucharest, comparing a pre-pandemic period (January 2019 to February 2020; *n* = 505) with the COVID-19 pandemic period (March 2020 to September 2022; *n* = 427). Across 932 non-duplicate blood culture isolates, our findings demonstrate a significant redistribution across microbial groups, a shift toward critical care settings, and substantial increases in resistance rates among Gram-negative organisms, against a backdrop of broad-spectrum antibiotic overuse and widespread empirical macrolide use. Glucose fermenters (Enterobacterales) decreased from 38.81% to 27.17% (*p* < 0.001), while glucose non-fermenters increased from 7.13% to 25.29% (*p* < 0.001), driven predominantly by *Acinetobacter baumannii*. Golli et al. (2025) similarly reported that more than 80% of their *A. baumannii* isolates were recovered during the post-pandemic period [14], which is comparable to our cohort, in which 80% of isolates were collected during the pandemic (pre-pandemic, *n* = 19; pandemic, *n* = 76). Also, the ECDC EARS-Net 2021 report documented a +43% rise in total isolations in 2021 versus 2018–2019 [15]. Streptococci isolated from blood cultures significantly decreased in the COVID-19 period compared with the pre-pandemic period, most probably because of the extensive and mandatory use of face masks during the COVID-19 period [16]. Yeasts emerged from complete absence to 4.45% of pandemic isolates (*p* < 0.001). The COVID-19 pandemic has been consistently associated with a marked global increase in *Candidozyma auris* colonization and infection, particularly among critically ill patients requiring intensive care. A systematic review and meta-analysis of 11 eligible studies estimated a pooled prevalence of *C. auris* infection of 13% (95% CI: 8–19%) among COVID-19 patients, with a pooled mortality rate of 37% (95% CI: 15–61%) [17]. This association has been attributed to the convergence of several risk factors characteristic of severe COVID-19, such as prolonged ICU stays, invasive mechanical ventilation, and broad-spectrum antibiotic and corticosteroid use, as well as disruption of routine infection-control practices under conditions of healthcare system strain. These factors facilitate both acquisition and nosocomial transmission of this multidrug-resistant yeast [18]. A descriptive study from US national surveillance data reported that clinical cases of *C. auris* in the United States increased every year since the first isolation, from a 44% increase in 2019 to a 95% increase during 2020–2021 [19]. Bloodstream infections represent a particularly severe manifestation of *C. auris* disease: in a large US multicenter analysis of 192 *C. auris*-associated hospitalizations, 38 (20%) involved bloodstream infection, with an associated crude mortality rate of 47% for bloodstream infections, compared to 31% for non-bloodstream infections [20]. Its first detection at our institution during the pandemic period, coinciding with the repurposing of the hospital for COVID-19 care and the associated increase in ICU admissions and prolonged critical illness, is consistent with these recognized risk factors. This finding reinforces the importance of continued species-level surveillance of yeast isolates and of maintaining heightened infection-control vigilance for this organism going forward.

The proportion of isolates from the Infectious Diseases ward fell from 86.34% to 53.63% (*p* < 0.001), while the ICU proportion more than tripled (10.50% → 32.32%, *p* < 0.001) because bacterial co-infection in SARS-CoV-2-positive patients was associated with more than usual ICU admission. Also, due to the fact that this hospital was a dedicated hospital for treating SARS-CoV-2-positive patients, Emergency Room isolates quadrupled proportionally (3.17% → 14.05%, *p* < 0.001).

Regarding AMR, for *E. coli*, our findings indicate that the pandemic period was associated with a numerical increase in antimicrobial resistance, particularly affecting third-generation cephalosporins, fluoroquinolones, and trimethoprim/sulfamethoxazole, although none of these increases remained statistically significant after correction for multiple comparisons. Resistance to carbapenems and last-line agents remained undetected throughout both periods. The pandemic-period Cefotaxime resistance of 23.08% exceeds the EU/EEA mean of 14.3% for third-generation cephalosporin resistance in *E. coli* recorded by ECDC EARS-Net 2022 [21], while our 0% carbapenem resistance is consistent with the EU mean of 0.3% reported for 2023 [22].

*Klebsiella pneumoniae* exhibited the most dramatic resistance escalation in our cohort, with statistically significant increases for nearly all antibiotic classes tested and ten of the eleven agents evaluated remaining significant after correction for multiple comparisons; only trimethoprim/sulfamethoxazole did not survive this correction (*q* = 0.120). The ECDC EARS-Net 2022 report recorded a nearly 50% increase in the EU/EEA incidence of carbapenem-resistant *K. pneumoniae* bloodstream infections between 2019 and 2022, reaching 10.9% overall [21]; our pandemic carbapenem resistance rates substantially exceed this figure (79.17% to MEM), closer to the resistance rate of Greece (72% in 2022) and more than Romania’s national data (47.8% in 2022) [7]. Our pandemic rates are likely to reflect the higher clinical severity and case mix of patients with culture-confirmed bloodstream infections treated at a tertiary care referral center. Golli et al. (2025) reported 64.15% carbapenem-resistant and 45.28% multidrug-resistant *K. pneumoniae* in an ICU of a hospital from Romania, with significant post-pandemic increases in Colistin (99.31%) resistance [14]. Our pandemic Colistin resistance in *K. pneumoniae* of 46.15% is substantially lower; this difference may be attributable to the fact that the isolates in the Golli et al. (2025) study [14] were collected exclusively from the Intensive Care Unit (ICU), whereas our isolates were recovered also from patients admitted to the emergency department and hospital wards. Petrakis et al. (2023) documented *K. pneumoniae* Colistin shifts over the same period similar to those from the Golli et al. (2025) study [14], in isolates recovered from an ICU of a Greek hospital (from 58.3% to 71.8%) [23].

*Acinetobacter baumannii* bloodstream isolates demonstrated significant increases in antimicrobial resistance during the pandemic for most tested antimicrobials, although only the increase in Colistin resistance remained significant after correction for multiple comparisons; the carbapenem findings, while directionally consistent with the 2021 ECDC EARS-Net report of a 121% increase in carbapenem-resistant *Acinetobacter* spp. [15], should accordingly be interpreted with some caution given our comparatively small pre-pandemic sample size (*n* = 19/505 pre-pandemic, vs. *n* = 76/427 in pandemic period). The Colistin resistance observed in our pandemic cohort (97.33%) closely paralleled Golli et al.’s post-pandemic rate (99.21%) [14]. This epidemiological shift towards the ICU (16.98% pre-pandemic to 28.99% in the pandemic of *A. baumannii* isolates) most plausibly reflects the changed case mix of the hospital during the pandemic period, when the institution functioned as a dedicated COVID-19 facility and the ICU population consisted largely of critically ill, mechanically ventilated patients with prolonged stays, which are established risk factors for *A. baumannii* acquisition and for selection of multidrug-resistant strains under sustained antimicrobial and biocide pressure. A contributing role for disruption to routine infection-control practices (e.g., cohorting, staffing ratios, environmental cleaning protocols) during pandemic surges, as documented at other institutions [24], cannot be excluded, though facility-level infection-control audit data were not available to quantify this contribution directly.

The wide variability in reported Colistin resistance rates during the pandemic period is illustrated by two other independent studies. Our Colistin resistance rates of 97.33% for *A. baumannii* and 46.15% for *K. pneumoniae* are consistent with the percentages reported in a dedicated referral setting ICU by Golli et al. (2025) (99.21% for *A. baumannii,* 99.31% for *K. pneumoniae*) [14] and in stark contrast with substantially lower rates in a general ICU cohort reported by Özçelik et al. (2026) in Türkiye (23.9% for *A. baumannii*, 32.1% for *K. pneumoniae*) [25]. The pattern of *A. baumannii* predominance among Colistin-resistant isolates during the pandemic period has also been independently reported at another tertiary hospital in Romania, further supporting the plausibility of our findings within the national context [26].

For both *Pseudomonas aeruginosa* and other Enterobacterales (*Enterobacter* spp. and miscellaneous species), no statistically significant changes in resistance rates were identified. This is consistent with the heterogeneous picture described by Reffat et al. (2024) [27], whose scoping review of 28 studies concluded that most pandemic-period AMR changes fell into the no-change or decrease categories. For *Pseudomonas aeruginosa,* 73% of the analyzed studies showed no change in resistance [27]. The lack of significance for *P. aeruginosa* in our study could be due to the relatively small sample size (*n* = 14 pre-pandemic, *n* = 25 pandemic).

Oxacillin resistance in *Staphylococcus aureus* (MRSA) bloodstream isolates decreased non-significantly post-pandemic (44.55% → 36.92%), consistent with the EU/EEA MRSA downward trend documented by ECDC EARS-Net 2022 [21], but the inducible Clindamycin resistance increased (RI CLI; 24.75% → 54.17%, *p* < 0.001), a finding that remained robust after correction for multiple comparisons, as also observed, though less robustly, in coagulase-negative staphylococci (13.89% → 40.48%, *p* = 0.012, *q =* 0.064). Strains that are carrying the iMLSB phenotype harbor *erm* genes (principally *ermA*, *ermB*, *ermC*, *ermTR*) that confer ribosomal methylation-mediated resistance to macrolides, lincosamides, and streptogramins [28]; the inducible phenotype appears susceptible on standard AST but expresses resistance following exposure to Clindamycin and is detected by the D-zone test [29]. Macrolide exposure preferentially selects for strains with inducible *erm* promoters over constitutive expression [28]; this mechanism explains the paradox of declining constitutive Clindamycin resistance alongside rising inducible resistance in our cohort. Whitaker et al. (2025) demonstrated marked increases in inducible MLS resistance in *S. aureus* from COVID-19 patients who received Azithromycin [30], and Glascock et al. (2026) showed that empirical Azithromycin in COVID-19 patients increased expression of *ermC*, *msrA*, and *ermX* within one day of treatment [31].

The dramatic pandemic-period increases in carbapenem resistance observed in our *K. pneumoniae* (CRKP) cohort (Imipenem 7.69% → 77.78% and Meropenem 15.38% → 79.17%) are consistent with surveillance data from another Bucharest Infectious Diseases hospital covering the same and immediately subsequent period. At the Victor Babeș Hospital, CRKP isolation rates increased to 37.4% in 2020, 40.0% in 2021, and 34.8% in 2022, rebounding post-pandemic to 45.81% in 2023 and 41.65% in 2024 [32]. Beyond the quantitative increase, a qualitative shift in carbapenemase types was documented, and OXA-48 predominated until 2020, but dual producers carrying NDM+OXA-48-like enzymes emerged in 2021 and became the dominant group by 2023–2024, displacing single OXA-48 and KPC producers [32]. In our study, the most frequent carbapenemase type in the pre-pandemic period was KPC (Table 4), similarly to the result of the 2019 CCRE survey —published as a landmark genomic study in 36 European countries—which confirmed the persistent spread of carbapenemase-producing *K. pneumoniae*, with KPC remaining dominant across *K. pneumoniae*. The most frequent carbapenemase genes identified were *bla*_KPC_, followed by *bla*_OXA-48-like_ and *bla*_NDM_, with new variants detected [33]. In the pandemic period, the NDM type of carbapenemase emerged amongst our *K. pneumoniae* strains and become dominant, followed (at a distance) by double carb apenemase (NDM+OXA-48-type), similarly to the study of Lazar DS et al. (2025) [32].

### Limitations of the Study

The retrospective single-center design limits generalizability. The pre-pandemic and pandemic observation windows differ in length (14 versus 31 months), introducing potential ascertainment bias. Several species had sample sizes too small for reliable antibiotic-level analysis—notably *A. baumannii* pre-pandemic (*n* = 19). Antimicrobial susceptibility was interpreted according to successive EUCAST breakpoint versions (9.0–12.0), which could have theoretically influenced susceptibility classification for selected organism–antimicrobial combinations. Although antimicrobial susceptibility was interpreted according to successive EUCAST breakpoint versions, the clinical MIC breakpoints for the carbapenems and Colistin remained unchanged across versions 9.0 through 12.0 for Enterobacterales, *Pseudomonas* spp., and *Acinetobacter* spp.; the observed resistance trends for these agents are therefore not attributable to changes in breakpoint classification during the study period. Despite the aforementioned limitations, the study provides locally relevant surveillance evidence applicable to empirical treatment decisions and antimicrobial stewardship at the institutional level.

## 5. Conclusions

For the analyzed period corresponding to the emergence of the COVID-19 pandemic, trends of both an increase and decrease in antibiotic resistance rates of bacterial strains isolated from blood cultures were observed. Most pathogens were more frequently isolated from ICU departments in the pandemic period, partially explainable by their origin from COVID-19 patients with bacterial co-infection or superinfection. There was a higher number of *Acinetobacter baumannii* and *Pseudomonas aeruginosa* strains isolated during the pandemic period, bacteria frequently associated with healthcare-associated infections and agents of ventilator-associated pneumonia. Additionally, there was a decreased number of *Escherichia coli* and streptococci strains isolated from blood cultures, which were more frequently encountered before the pandemic period of the study.

The study provides insights into the dynamic nature of antibiotic resistance in an Infectious Diseases hospital during the COVID-19 pandemic and the period before. By identifying shifting trends in the type of microorganisms and resistance patterns, it contributes to understanding the complex interplay between viral and bacterial co-infection and antimicrobial resistance, ultimately informing strategies to combat both public health threats.

## Figures and Tables

**Table 1:** Microorganisms isolated from blood cultures during the pre-pandemic and pandemic periods, by hospital ward (Infectious Diseases, Emergency Room, and Intensive Care Unit).

The Microorganisms	Pre-Pandemic			Pandemic			Total
	ID	ER	ICU	ID	ER	ICU	
*Escherichia coli*	113	2	2	29	6	4	156
*Klebsiella pneumoniae*	37	2	2	16	4	28	89
*Enterobacter* spp.	11	1	1	2	2	3	20
Other Enterobacterales	20	1	4	13	4	5	47
*Pseudomonas aeruginosa*	10	1	3	9	2	14	39
*Acinetobacter baumannii*	8	2	9	29	7	40	95
*Burkholderia cepacia*	2	0	0	0	0	0	2
*Stenotrophomonas maltophilia*	0	1	0	1	0	5	7
*Delftia acidovorans*	0	0	0	0	0	1	1
*Staphylococcus aureus*	84	4	13	56	10	6	173
Coagulase-negative staphylococci	32	0	4	19	10	13	78
*Enterococcus faecalis*	14	0	4	17	1	2	38
*Enterococcus faecium*	10	0	0	2	1	2	15
*Streptococcus pneumoniae*	21	1	6	4	1	4	37
*Streptococcus viridans*	33	1	3	14	3	0	54
*Streptococcus β-* *haemolytic*	27	0	2	9	3	0	41
*Bacteroides fragilis*	6	0	0	1	1	0	8
*Clostridium perfringens*	2	0	0	1	0	0	3
*Fusobacterium necrophorum*	2	0	0	0	0	0	2
*Neisseria meningitidis*	2	0	0	0	0	0	2
*Listeria monocytogenes*	1	0	0	1	0	0	2
*Granulicatella adiacens*	1	0	0	0	0	0	1
*Hafnia alvei*	0	0	0	1	0	0	1
*Elizabethkingia miricola*	0	0	0	0	0	1	1
*Aggregatibacter aphrophilus*	0	0	0	1	0	0	1
*Candida albicans*	0	0	0	0	2	3	5
*Candida tropicalis*	0	0	0	0	0	1	1
*Candida glabrata*	0	0	0	0	1	0	1
*Candida krusei*/*Issatchenchia orientalis*	0	0	0	0	1	0	1
*Candida parapsilosis*	0	0	0	3	1	6	10
*Candidozyma auris*	0	0	0	1	0	0	1
TOTAL	436	16	53	229	60	138	932

Note: ID—Infectious Diseases Ward, ER—Emergency Room, ICU—Intensive Care Unit.

**Table 2:** The difference in isolation of the blood culture microorganisms during the pre-pandemic and pandemic periods by ward.

Ward	Pre-Pandemic		Pandemic		*p*-Value
	*n*	%	*n*	%	
ID	436	86.34	229	53.63	**<0.001**
ER	16	3.17	60	14.05	**<0.001**
ICU	53	10.50	138	32.32	**<0.001**

Note: n—Number, ID—Infectious Diseases, ER—Emergency Room, ICU—Intensive Care Unit; values in bold are statistically significant.

**Table 3:** Microbial group change between pre-pandemic and pandemic period.

Microbial Group	Pre-Pandemic		Pandemic		*p*-Value
	*n*	%	*n*	%	
Glucose fermenters	196	38.81	116	27.17	**<0.001**
Glucose non-fermenters	36	7.13	108	25.29	**<0.001**
*Staphylococcus* spp.	137	27.13	114	26.70	0.9409
*Streptococcus* spp.	94	18.61	38	8.90	**<0.001**
*Enterococcus* spp.	28	5.55	25	5.85	0.8876
Anaerobic bacteria	10	1.98	3	0.7	0.1589
Other bacteria	4	0.79	4	0.94	1.0000
Yeasts	0	0.00	19	4.45	**<0.001**

Note: n—number; values in bold are statistically significant.

**Table 4:** Carbapenemases in *K. pneumoniae* in the pre-pandemic and pandemic periods.

Carbapenemase	Pre-Pandemic	Pandemic
KPC	5	10
OXA-48-type	1	3
NDM	1	21
NDM + OXA-48-type	0	4
KPC + NDM + OXA-48-type	0	1
Total	7	39

## Data Availability

The original contributions presented in this study are included in the article/Supplementary Material. Further inquiries can be directed to the corresponding author.

## Supplementary Materials

The following supporting information can be downloaded at https://www.mdpi.com/article/10.3390/microorganisms14102269/s1, Table S1: Antimicrobial resistance rates, 95% confidence intervals, and FDR-corrected significance, pre-pandemic vs. pandemic period.

## Abbreviations

The following abbreviations are used in this manuscript:

AMR	Antimicrobial resistance
AST	Antimicrobial susceptibility testing
MIC	Minimum inhibitory concentration
MRSA	Methicillin-resistant *Staphylococcus aureus*
ESBL	Extended-spectrum beta-lactamase
MDR	Multidrug-resistant
ID	Infectious diseases
ER	Emergency room
ICU	Intensive care unit
CIP	Ciprofloxacin
TCY	Tetracycline
VAN	Vancomycin
LNZ	Linezolid
AMC	Amoxicillin/clavulanic acid
TZP	Piperacillin-tazobactam
CRO/CAZ	Ceftriaxone/ceftazidime
MEM	Meropenem
COL	Colistin
GEN	Gentamicin
FEP	Cefepime
AMK	Amikacin
TOB	Tobramycin
FQ	Fluoroquinolones
IPM	Imipenem
