# Supplementary material for: Changes in Antimicrobial Resistance in Bloodstream Infections During the COVID-19 Pandemic—A Retrospective Study from a Tertiary Care Hospital
Source: Microorganisms. 2026 Oct 6;14(10):2269. doi: 10.3390/microorganisms14102269 (PMC13707747; doi:10.3390/microorganisms14102269)
Supplement: Supplementary file 1 [file microorganisms-14-02269-s001.zip › microorganisms-4506109-supplementary.pdf]

Supplementary Table S1. Antimicrobial resistance rates, 95% confidence intervals, and FDR-corrected significance, pre-pandemic vs. pandemic period

Organism	Antibiotic	N (pre)	R (pre)	% R (pre)	95% CI (pre)	N (pandemic)	R (pandemic)	% R (pandemic)	95% CI (pandemic)	*p*-value	*q*-value (FDR)
Escherichia coli	Ampicillin	117	79	67.52	58.6–75.3	39	32	82.05	67.3–91.0	0.103	0.273
Escherichia coli	Amoxicillin/clavulanate	115	31	26.96	19.7–35.7	39	12	30.77	18.6–46.4	0.682	0.927
Escherichia coli	Piperacillin/tazobactam	116	9	7.76	4.1–14.1	38	3	7.89	2.7–20.8	1.000	1.000
Escherichia coli	Cefotaxime	117	19	16.24	10.6–24.0	39	9	23.08	12.6–38.3	0.343	0.653
Escherichia coli	Ceftazidime	117	14	11.97	7.3–19.1	39	9	23.08	12.6–38.3	0.117	0.290
Escherichia coli	Cefepime	115	13	11.30	6.7–18.4	39	7	17.95	9.0–32.7	0.283	0.594
Escherichia coli	Amikacin	117	1	0.85	0.2–4.7	39	0	0.00	0.0–9.0	1.000	1.000
Escherichia coli	Gentamicin	117	9	7.69	4.1–14.0	39	1	2.56	0.5–13.2	0.453	0.825
Escherichia coli	Ciprofloxacin	117	19	16.24	10.6–24.0	39	11	28.21	16.5–43.8	0.107	0.274
Escherichia coli	Imipenem	117	0	0.00	0.0–3.2	38	0	0.00	0.0–9.2	–	–
Escherichia coli	Meropenem	117	0	0.00	0.0–3.2	39	0	0.00	0.0–9.0	–	–
Escherichia coli	Tigecycline	117	0	0.00	0.0–3.2	39	0	0.00	0.0–9.0	–	–
Escherichia coli	Trimethoprim/sulfamethoxazole	116	36	31.03	23.3–39.9	39	20	51.28	36.2–66.1	0.033	0.127
Escherichia coli	Colistin	79	0	0.00	0.0–4.6	13	0	0.00	0.0–22.8	–	–
Klebsiella pneumoniae	Amoxicillin/clavulanate	41	13	31.71	19.6–47.0	44	38	86.36	73.3–93.6	**<0.001**	<0.001
Klebsiella pneumoniae	Cefotaxime	40	12	30.00	18.1–45.4	44	39	88.64	76.0–95.0	**<0.001**	<0.001
Klebsiella pneumoniae	Ceftazidime	40	12	30.00	18.1–45.4	47	41	87.23	74.8–94.0	**<0.001**	<0.001
Klebsiella pneumoniae	Cefepime	40	12	30.00	18.1–45.4	47	40	85.11	72.3–92.6	**<0.001**	<0.001
Klebsiella pneumoniae	Amikacin	41	2	4.88	1.3–16.1	48	34	70.83	56.8–81.8	**<0.001**	<0.001
Klebsiella pneumoniae	Gentamicin	40	7	17.50	8.7–31.9	46	33	71.74	57.5–82.7	**<0.001**	<0.001
Klebsiella pneumoniae	Ciprofloxacin	40	13	32.50	20.1–48.0	47	40	85.11	72.3–92.6	**<0.001**	<0.001
Klebsiella pneumoniae	Imipenem	39	3	7.69	2.7–20.3	45	35	77.78	63.7–87.5	**<0.001**	<0.001
Klebsiella pneumoniae	Meropenem	39	6	15.38	7.2–29.7	48	38	79.17	65.7–88.3	**<0.001**	<0.001
Klebsiella pneumoniae	Trimethoprim/sulfamethoxazole	39	11	28.21	16.5–43.8	47	25	53.19	39.2–66.7	0.028	0.120
Klebsiella pneumoniae	Colistin	27	3	11.11	3.9–28.1	26	12	46.15	28.8–64.5	**0.006**	0.043
Other Enterobacterales	Piperacillin/tazobactam	35	3	8.57	3.0–22.4	28	4	14.29	5.7–31.5	0.690	0.927
Other Enterobacterales	Cefotaxime	35	8	22.86	12.1–39.0	28	8	28.57	15.3–47.1	0.772	0.974
Other Enterobacterales	Ceftazidime	37	8	21.62	11.4–37.2	27	6	22.22	10.6–40.8	1.000	1.000
Other Enterobacterales	Cefepime	38	5	13.16	5.8–27.3	28	4	14.29	5.7–31.5	1.000	1.000
Other Enterobacterales	Amikacin	38	2	5.26	1.5–17.3	28	2	7.14	2.0–22.6	1.000	1.000
Other Enterobacterales	Ciprofloxacin	34	12	35.29	21.5–52.1	29	13	44.83	28.4–62.5	0.606	0.920
Other Enterobacterales	Imipenem	37	1	2.70	0.5–13.8	23	3	13.04	4.5–32.1	0.153	0.357
Other Enterobacterales	Meropenem	38	1	2.63	0.5–13.5	28	2	7.14	2.0–22.6	0.570	0.915
Other Enterobacterales	Trimethoprim/sulfamethoxazole	37	6	16.22	7.7–31.1	28	7	25.00	12.7–43.4	0.533	0.910
Acinetobacter baumannii	Amikacin	19	9	47.37	27.3–68.3	73	56	76.71	65.8–84.9	0.022	0.105
Acinetobacter baumannii	Tobramycin	17	9	52.94	31.0–73.8	71	53	74.65	63.4–83.3	0.136	0.328

Organism	Antibiotic	N (pre)	R (pre)	% R (pre)	95% CI (pre)	N (pandemic)	R (pandemic)	% R (pandemic)	95% CI (pandemic)	*p*-value	*q*-value (FDR)
Acinetobacter baumannii	Levofloxacin	16	13	81.25	57.0–93.4	76	74	97.37	90.9–99.3	0.035	0.127
Acinetobacter baumannii	Imipenem	19	15	78.95	56.7–91.5	73	70	95.89	88.6–98.6	0.031	0.127
Acinetobacter baumannii	Meropenem	19	15	78.95	56.7–91.5	76	74	97.37	90.9–99.3	0.014	0.075
Acinetobacter baumannii	Trimethoprim/sulfamethoxazole	17	13	76.47	52.7–90.4	65	61	93.85	85.2–97.6	0.054	0.183
Acinetobacter baumannii	Colistin	19	0	0.00	0.0–16.8	75	73	97.33	90.8–99.3	**<0.001**	<0.001
Pseudomonas aeruginosa	Piperacillin/tazobactam	14	3	21.43	7.6–47.6	23	9	39.13	22.2–59.2	0.306	0.628
Pseudomonas aeruginosa	Ceftazidime	14	3	21.43	7.6–47.6	25	8	32.00	17.2–51.6	0.713	0.928
Pseudomonas aeruginosa	Cefepime	14	4	28.57	11.7–54.6	25	7	28.00	14.3–47.6	1.000	1.000
Pseudomonas aeruginosa	Amikacin	14	0	0.00	0.0–21.5	25	6	24.00	11.5–43.4	0.071	0.222
Pseudomonas aeruginosa	Tobramycin	13	2	15.38	4.3–42.2	24	6	25.00	12.0–44.9	0.685	0.927
Pseudomonas aeruginosa	Ciprofloxacin	14	6	42.86	21.4–67.4	22	8	36.36	19.7–57.0	0.738	0.945
Pseudomonas aeruginosa	Imipenem	14	5	35.71	16.3–61.2	25	8	32.00	17.2–51.6	1.000	1.000
Pseudomonas aeruginosa	Meropenem	14	4	28.57	11.7–54.6	25	7	28.00	14.3–47.6	1.000	1.000
Pseudomonas aeruginosa	Colistin	14	0	0.00	0.0–21.5	24	1	4.17	0.7–20.2	1.000	1.000
Staphylococcus aureus	Penicillin	99	80	80.81	72.0–87.4	71	58	81.69	71.2–89.0	1.000	1.000
Staphylococcus aureus	Oxacillin	101	45	44.55	35.2–54.3	65	24	36.92	26.2–49.1	0.339	0.653
Staphylococcus aureus	Erythromycin	100	51	51.00	41.3–60.6	72	40	55.56	44.1–66.5	0.643	0.927
Staphylococcus aureus	Clindamycin	101	5	4.95	2.1–11.1	72	2	2.78	0.8–9.6	0.701	0.927
Staphylococcus aureus	Clindamycin (inducible resistance)	101	25	24.75	17.4–34.0	72	39	54.17	42.7–65.2	**<0.001**	<0.001
Staphylococcus aureus	Gentamicin	100	4	4.00	1.6–9.8	72	2	2.78	0.8–9.6	1.000	1.000
Staphylococcus aureus	Ciprofloxacin	100	16	16.00	10.1–24.4	59	2	3.39	0.9–11.5	0.018	0.094
Staphylococcus aureus	Moxifloxacin	95	10	10.53	5.8–18.3	72	1	1.39	0.2–7.5	0.025	0.112
Staphylococcus aureus	Tetracycline	94	43	45.74	36.0–55.8	72	34	47.22	36.1–58.6	0.876	1.000
Staphylococcus aureus	Tigecycline	87	0	0.00	0.0–4.2	66	1	1.52	0.3–8.1	0.431	0.804
Staphylococcus aureus	Trimethoprim/sulfamethoxazole	96	3	3.12	1.1–8.8	72	0	0.00	0.0–5.1	0.261	0.578
Staphylococcus aureus	Linezolid	101	0	0.00	0.0–3.7	71	0	0.00	0.0–5.1	–	–
Staphylococcus aureus	Teicoplanin	98	0	0.00	0.0–3.8	72	0	0.00	0.0–5.1	–	–
Staphylococcus aureus	Vancomycin	100	0	0.00	0.0–3.7	71	0	0.00	0.0–5.1	–	–
Coagulase-negative staphylococci	Oxacillin	35	31	88.57	74.0–95.5	37	26	70.27	54.2–82.5	0.082	0.248
Coagulase-negative staphylococci	Erythromycin	36	27	75.00	58.9–86.2	42	23	54.76	39.9–68.8	0.097	0.273
Coagulase-negative staphylococci	Clindamycin	36	10	27.78	15.8–44.0	42	9	21.43	11.7–35.9	0.601	0.920
Coagulase-negative staphylococci	Clindamycin (inducible resistance)	36	5	13.89	6.1–28.7	42	17	40.48	27.0–55.5	0.012	0.069
Coagulase-negative staphylococci	Gentamicin	36	15	41.67	27.1–57.8	42	14	33.33	21.0–48.4	0.488	0.853
Coagulase-negative staphylococci	Ciprofloxacin	35	21	60.00	43.6–74.4	34	19	55.88	39.5–71.1	0.809	1.000
Coagulase-negative staphylococci	Moxifloxacin	32	17	53.12	36.4–69.1	42	14	33.33	21.0–48.4	0.101	0.273
Coagulase-negative staphylococci	Tetracycline	29	17	58.62	40.7–74.5	42	19	45.24	31.2–60.1	0.337	0.653
Coagulase-negative staphylococci	Trimethoprim/sulfamethoxazole	35	8	22.86	12.1–39.0	40	7	17.50	8.7–31.9	0.579	0.915
Coagulase-negative staphylococci	Linezolid	36	4	11.11	4.4–25.3	41	3	7.32	2.5–19.4	0.699	0.927
Coagulase-negative staphylococci	Vancomycin	34	0	0.00	0.0–10.2	39	0	0.00	0.0–9.0	–	–

Organism	Antibiotic	N (pre)	R (pre)	% R (pre)	95% CI (pre)	N (pandemic)	R (pandemic)	% R (pandemic)	95% CI (pandemic)	*p*-value	*q*-value (FDR)
Streptococcus pneumoniae	Penicillin	28	4	14.29	5.7–31.5	9	1	11.11	2.0–43.5	1.000	1.000
Streptococcus pneumoniae	Ampicillin	27	4	14.81	5.9–32.5	9	2	22.22	6.3–54.7	0.627	0.927
Streptococcus pneumoniae	Cefotaxime	27	2	7.41	2.1–23.4	9	0	0.00	0.0–29.9	1.000	1.000
Streptococcus pneumoniae	Ceftriaxone	25	1	4.00	0.7–19.5	9	0	0.00	0.0–29.9	1.000	1.000
Streptococcus pneumoniae	Erythromycin	28	8	28.57	15.3–47.1	9	6	66.67	35.4–87.9	0.057	0.187
Streptococcus pneumoniae	Clindamycin	28	5	17.86	7.9–35.6	9	6	66.67	35.4–87.9	0.011	0.067
Streptococcus pneumoniae	Moxifloxacin	28	0	0.00	0.0–12.1	9	0	0.00	0.0–29.9	–	–
Streptococcus pneumoniae	Tetracycline	28	6	21.43	10.2–39.5	9	6	66.67	35.4–87.9	0.036	0.127
Streptococcus pneumoniae	Trimethoprim/sulfamethoxazole	28	10	35.71	20.7–54.2	9	2	22.22	6.3–54.7	0.687	0.927
Streptococcus pneumoniae	Linezolid	28	0	0.00	0.0–12.1	9	0	0.00	0.0–29.9	–	–
Streptococcus pneumoniae	Teicoplanin	28	0	0.00	0.0–12.1	9	0	0.00	0.0–29.9	–	–
Streptococcus pneumoniae	Vancomycin	25	0	0.00	0.0–13.3	9	0	0.00	0.0–29.9	–	–
Enterococcus faecalis	Gentamicin (high-level)	18	6	33.33	16.3–56.3	18	6	33.33	16.3–56.3	1.000	1.000
Enterococcus faecalis	Streptomycin (high-level)	18	5	27.78	12.5–50.9	18	8	44.44	24.6–66.3	0.489	0.853
Enterococcus faecalis	Tigecycline	12	0	0.00	0.0–24.2	20	0	0.00	0.0–16.1	–	–
Enterococcus faecalis	Teicoplanin	17	0	0.00	0.0–18.4	20	0	0.00	0.0–16.1	–	–
Enterococcus faecalis	Vancomycin	18	0	0.00	0.0–17.6	20	0	0.00	0.0–16.1	–	–
Enterococcus faecium	Gentamicin (high-level)	10	7	70.00	39.7–89.2	4	2	50.00	15.0–85.0	0.580	0.915
Enterococcus faecium	Streptomycin (high-level)	10	8	80.00	49.0–94.3	5	2	40.00	11.8–76.9	0.251	0.571
Enterococcus faecium	Tigecycline	9	0	0.00	0.0–29.9	5	0	0.00	0.0–43.4	–	–
Enterococcus faecium	Teicoplanin	10	6	60.00	31.3–83.2	5	1	20.00	3.6–62.4	0.282	0.594
Enterococcus faecium	Vancomycin	10	8	80.00	49.0–94.3	5	1	20.00	3.6–62.4	0.089	0.260

Note: To account for multiple testing, p-values from Fisher's exact test were adjusted using the Benjamini–Hochberg false discovery rate (FDR) procedure. Of the 98 microorganism–antimicrobial combinations analysed, 16 showed no resistant isolates in either period, so no statistical comparison was applicable; the correction was therefore applied to the $m = 82$ tests actually performed. Adjusted q-values below 0.05 were considered statistically significant.
